# A randomized wait-list controlled trial of a social support intervention for caregivers of patients with primary malignant brain tumor

**DOI:** 10.1186/s12913-021-06372-w

**Published:** 2021-04-17

**Authors:** Maija Reblin, Dana Ketcher, Rachael McCormick, Veronica Barrios-Monroy, Steven K. Sutton, Bradley Zebrack, Kristen J. Wells, Solmaz Sahebjam, Peter Forsyth, Margaret M. Byrne

**Affiliations:** 1 Department of Health Outcomes & Behavior, Moffitt Cancer Center, 12902 Magnolia Dr, Tampa, FL 33612 USA; 2 Department of Biostatistics and Bioinformatics, Moffitt Cancer Center, Tampa, FL USA; 3 School of Social Work, University of Michigan, Ann Arbor, MI USA; 4 Department of Psychology, San Diego State University, San Diego, CA USA; 5 SDSU/UC San Diego Joint Doctoral Program in Clinical Psychology, San Diego, CA USA; 6 Department of Neuro-Oncology, Moffitt Cancer Center, Tampa, FL USA

**Keywords:** Caregiver, Oncology, Social support, Psychosocial intervention, Clinical navigation, mHealth

## Abstract

**Background:**

Informal family caregivers constitute an important and increasingly demanding role in the cancer healthcare system. This is especially true for caregivers of patients with primary malignant brain tumors based on the rapid progression of disease, including physical and cognitive debilitation. Informal social network resources such as friends and family can provide social support to caregivers, which lowers caregiver burden and improves overall quality of life. However, barriers to obtaining needed social support exist for caregivers. To address this need, our team developed and is assessing a multi-component caregiver support intervention that uses a blend of technology and personal contact to improve caregiver social support.

**Methods:**

We are currently conducting a prospective, longitudinal 2-group randomized controlled trial which compares caregivers who receive the intervention to a wait-list control group. Only caregivers directly receive the intervention, but the patient-caregiver dyads are enrolled so we can assess outcomes in both. The 8-week intervention consists of two components: (1) The electronic Social Network Assessment Program, a web-based tool to visualize existing social support resources and provide a tailored list of additional resources; and (2) Caregiver Navigation, including weekly phone sessions with a Caregiver Navigator to address caregiver social support needs. Outcomes are assessed by questionnaires completed by the caregiver (baseline, 4-week, 8-week) and the cancer patient (baseline, and 8-week). At 8 weeks, caregivers in the wait-list condition may opt into the intervention. Our primary outcome is caregiver well-being; we also explore patient well-being and caregiver and patient health care utilization.

**Discussion:**

This protocol describes a study testing a novel social support intervention that pairs a web-based social network visualization tool and resource list (eSNAP) with personalized caregiver navigation. This intervention is responsive to a family-centered model of care and calls for clinical and research priorities focused on informal caregiving research.

**Trial registration:**

clinicaltrials.gov, Registration number: NCT04268979; Date of registration: February 10, 2020, retrospectively registered.

## Background

Caregiving is an important public health priority [1, 2] and the role of informal family caregivers in the health care system is expanding [3]. Family caregivers are defined by the American Cancer Society as individuals who help or support a person with cancer and are not paid to do so; often, this role is taken on by family members or close friends (“chosen family”; [4]). These caregivers relieve demands on the formal health care system by performing care tasks and help patients remain at home [5, 6]. However, informal caregiving can be burdensome [7–9] and adversely affect caregiver well-being [10–12], which is associated with higher patient distress, increased risk/rate of hospitalization, and excess mortality [13–15].

As described in the Stress Process Model of Caregiving [16], social support and other means of coping can impact the relationship between caregiving burden and well-being. Caregivers whose informal social network (friends, family, acquaintances) provides adequate social support experience lower levels of burden, better health, and improved quality of life [17–21]. This support can also positively impact patients, whose psychosocial outcomes are highly interdependent with caregivers’ [22–24]. Caregivers with resources to cope, conferred through social support, are also better able to keep patients at home, thus decreasing costly hospitalizations [13, 25–27]. However, caregivers experience barriers to obtaining support, including intrapersonal barriers, such as difficulty identifying available support in the moment, interpersonal barriers, such as difficulty asking for help, and systemic barriers, such as a lack of availability of formal, comprehensive, proactive caregiver support programs [20, 28–30]. As a result, caregivers may not receive any support or may receive support too late to benefit [31, 32].

Caregivers of patients with a primary malignant brain tumor face unique demands and increased burden [33–35] based on rapid disease progression, significant physical debilitation, cognitive decline, as well as personality and behavior changes associated with the disease [33, 36–39]. However, despite their potentially greater need, few support interventions have been specifically designed for and assessed in caregivers of patients with primary brain tumor [40].

**Fig. 1:**
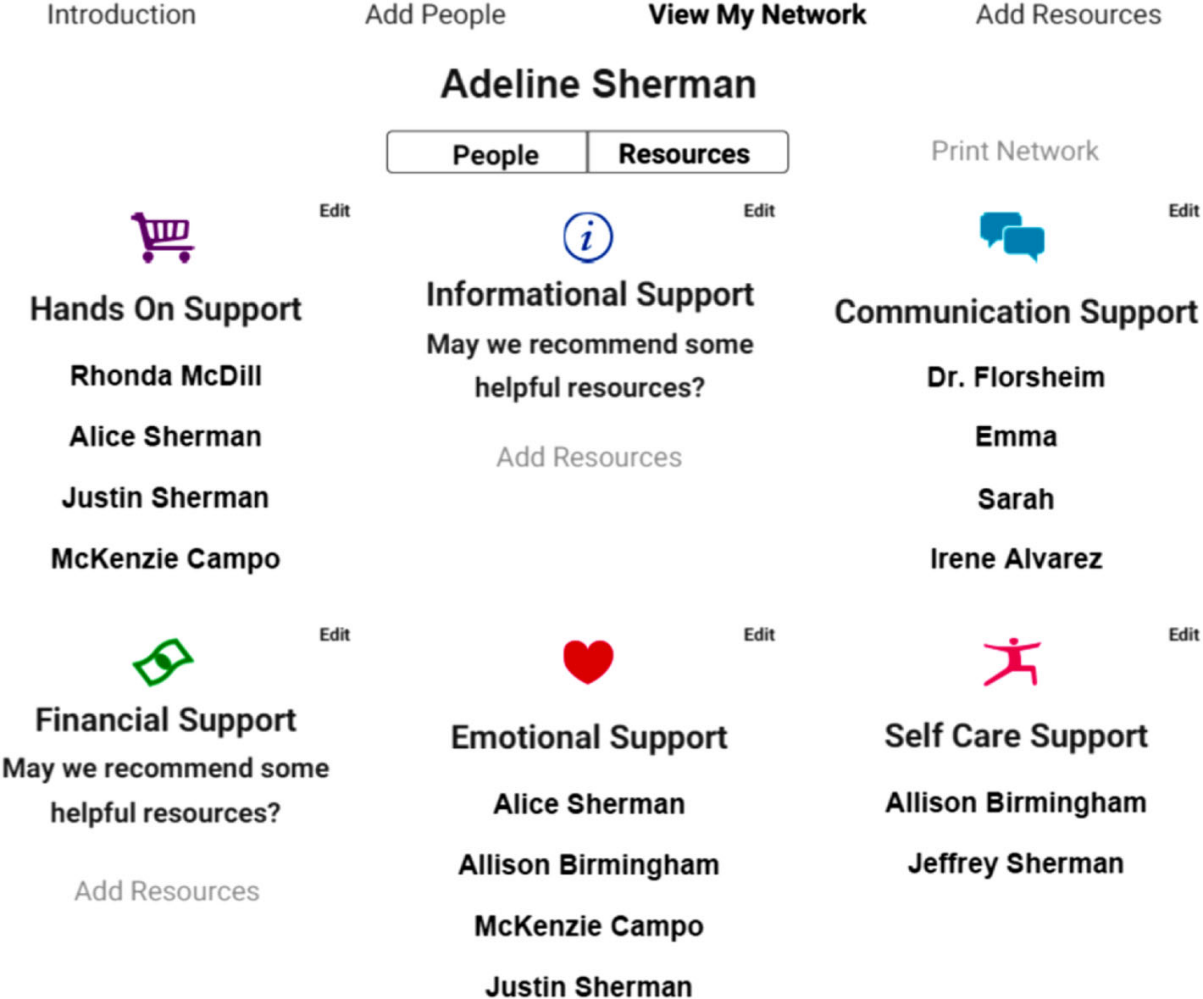
Example eSNAP screen

To overcome barriers in caregivers’ social support utilization, our team has developed a caregiver support intervention with input from caregivers of patients with primary malignant brain tumor. The 8-week intervention blends technology with personal contact, and consists of two components. The first component is the electronic Social Network Assessment Program (eSNAP), a web-based tool to visualize existing social support resources and provide a tailored list of additional resources [30, 41]. As seen in Fig. 1, eSNAP quickly collects and organizes social support information entered by caregivers to visualize the size, quality, and function of support networks. Visualizations can help caregivers catalogue support resources and present them in a new way, which may make them more salient and remind caregivers of their availability. The second component of the intervention is assistance from a Caregiver Navigator. The Caregiver Navigator assesses social support via eSNAP and provides navigation via brief, weekly telephone sessions. Navigation sessions are designed to help caregivers identify and problem-solve barriers to finding support resources to meet their needs, including help with identifying and leveraging support within their own informal social network (i.e. from family/friends). These sessions are also intended to enroll or direct caregivers to formal services within the institution or community (e.g. social work, mentorship).

## Methods/design

### Study aims

The primary aim of this randomized controlled trial (RCT) is to evaluate the efficacy of the eSNAP + Caregiver Navigator intervention on caregiver wellbeing. We hypothesize caregivers of patients with primary malignant brain tumor who receive the intervention will report less perceived burden, operationalized as scores on the Zarit Burden Scale Short Form [42], and lower levels of distress, operationalized as scores on the Patient Health Questionnaire-8 (PHQ-8) [43] and the General Anxiety Disorder-7 (GAD-7) [44], when compared with waitlist controls at the Week 8 follow-up. Although the intervention is caregiver-focused, given the interdependent relationship between caregiver and patient, we anticipate our intervention will also indirectly impact patient well-being and health care utilization outcomes. Thus, our second aim is to evaluate the efficacy of eSNAP + Navigator on patient well-being and patient and caregiver health care utilization. We hypothesize that patients whose caregivers receive the intervention will report less distress, operationalized as scores on the PHQ-8 and GAD-7, lower numbers of patient unplanned outpatient appointments, ER visits, and hospitalizations, and increased caregiver use of social work or other support services, when compared with waitlist controls at the Week 8 follow-up.

### Study design

**Fig. 2:**
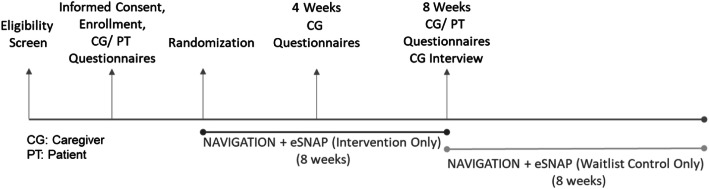
Study Schema

Our study uses a prospective, longitudinal 2-group RCT design, comparing participants who receive the intervention to a wait-list control group (Fig. 2). The COVID-19 pandemic has impacted the ability of study team members to recruit potential participants, due to limits on non-essential staff and visitors allowed in clinic for in-person recruitment as well as lower clinic volumes. However, the key study activities are conducted online (data collection, eSNAP) and by phone (Caregiver Navigation), and as such, the protocol has not significantly been changed by the pandemic.

### Participants

Participant inclusion criteria include: 1) age 21+ years, 2) English-speaking, and 3) able to complete questionnaires (including by proxy). Caregivers must self-identify as being a primary caregiver—a family member, friend, or other unpaid person—who provides at least some care at home for a patient with primary malignant brain tumor. Patients must be diagnosed with a new or recurrent primary malignant brain tumor within the last 6 months, be undergoing active treatment, and have a prognosis of at least 6 months. Both patients and caregivers must consent to study participation.

### Recruitment

Participants are recruited through the Moffitt Cancer Center Neuro-Oncology Clinic. Patients and accompanying caregivers are approached during scheduled clinic visits and/or mailed study information around the time of a clinic visit. Flyers also are available in clinic for oncologists and other staff to distribute. Study staff meet in person or by phone with caregivers and patients who express interest in the study to provide additional information and answer any questions before obtaining verbal informed consent, which is documented by research staff.

### Procedures

Caregiver and patient dyads are recruited within 6 months of patient diagnosis. At enrollment, participants complete baseline questionnaires electronically using REDCap (paper versions will be available upon request). Caregiver questionnaires include demographics, burden, social support, and health care utilization measures. Patient questionnaires include measures of distress. Upon completion of questionnaires, caregiver participants are randomized by study staff to either the intervention (use of eSNAP + Caregiver Navigator) or waitlist control condition. Participants are randomized by study staff to either the intervention (use of eSNAP + Caregiver Navigator) or wait list control conditions. Participants are randomized using an automated random assignment tool in REDCap (Research Electronic Data Capture; [45, 46]), a secure data collection and management software. To ensure temporal balance between conditions, a randomized block design (blocks of 8) is used and participants are stratified by caregiver sex. Condition is not blinded.

### Waitlist control

Participants randomly assigned to the waitlist control condition complete questionnaires during the 8-week study period. After the 8 weeks, they are invited to access the intervention as described below, including completion of questionnaires, eSNAP, and 8 weeks of Caregiver Navigator sessions. A waitlist control condition was selected to capture usual care conditions and to avoid potential confounding of the additional social support that often accompanies an attention-control condition. Waitlisted participants, as in usual care, may engage their own support networks and seek or get referrals to formal support services from health care providers as needed. However, waitlisted participants receive no social network visualization and there is no existing process in place to proactively and systematically direct caregivers to engage informal or formal support resources.

### eSNAP and caregiver navigation

Caregivers randomly assigned to the intervention condition receive access to eSNAP and Caregiver Navigation sessions. First, caregivers are enrolled in eSNAP. Caregivers are assigned a user name and password and instructed on how to complete their support visualization in eSNAP using a personal device. In addition to a visualization of existing support, eSNAP makes tailored suggestions for additional resources available within the cancer center or community. Study staff are available to oversee the eSNAP process and answer questions. If participants are not able to complete eSNAP in one attempt, they may save their input and return to it later. Intervention participants are able to access and edit their eSNAP visualization or access resources lists at any time (with reminders given weekly during navigation). To assist enrolled caregivers without internet access at home, tablets are available to study participants to use while at Moffitt at any point during the study period.

Second, caregivers receive sessions from a Caregiver Navigator. Two Caregiver Navigators with non-clinical background were provided education on the neuro-oncology patient and caregiver experience, including reading and didactic sessions, shadowing, and informant interviews. Navigators reviewed available support resources collected by the study team and conducted a scan for additional resources in the cancer center and community. Training was also provided on key navigation skills, including assessment, problem-solving, and motivational interviewing, as well as basic research skills and ethics. Upon the first contact, the Caregiver Navigator conducts an intake assessment the caregiver’s social support and burden based on the eSNAP support visualization and through telephone consultation. Based on this assessment, the Caregiver Navigator develops a caregiver-specific plan to address caregiver support needs over the additional 8 navigator sessions (See Table 1). The Caregiver Navigator may provide social support directly (e.g., emotional, informational) or may assist caregivers in obtaining support resources from their network or from formal resources through motivational interviewing or problem solving.

**Table 1:** Caregiver Navigator session topics

Topic	Description
Overview	Introduction to caregiver navigation, setting goals/expectations
Getting social support	Review of existing social support resources and needs, identifying facilitators and barriers to social support
Problem solving	Introduction of step-wise process of identifying a problem, generating solutions, including identifying support, planning steps towards action
Growing your social support network	Review of potential additional/overlooked support from informal/formal sources
How to ask for help	Review best practices and rehearse asking for help
Taking care of yourself	Review importance of self-care and identify resources to facilitate self-care activities
Setting limits and dealing with unhelpful support	Review importance and rehearse strategies to implement boundaries to reduce social stress
Positive side of caregiving	Review caregiver achievements and growth
Review and planning for the future	Reflection on previous sessions, potential gains, and identify strategies to continue practicing

Navigation calls are anticipated to be approximately 30 min weekly over 8 weeks. Caregivers are also able to contact navigators outside of session if needed. Each session begins with a brief assessment and manualized topics are planned, but sessions vary depending on caregiver needs. The navigator is flexible given dynamic changes that occur with each patient’s functioning, treatment, and prognosis, and caregiver’s needs and resources to handle changes. A major goal of the Caregiver Navigator is to provide caregivers with tools to identify and capitalize on their existing support resources and integrate them with available formal services, including Moffitt social work. This integration helps to transition caregivers from the Caregiver Navigator at the end of the study period. However, this transition may be delayed if caregivers are in crisis at the end of 8 weeks.

A log is made of all Caregiver Navigator contacts, including date/time, caregiver needs and barriers to obtaining support, and the actions taken by the navigator to meet needs or overcome barriers [47]. This log will also be valuable in determining the “active ingredient” or most common/most effective Caregiver Navigator activities. Caregiver Navigator sessions may be recorded (with participant permission) for training or fidelity purposes.

All caregiver participants are asked to complete follow-up questionnaires by emailed or mailed surveys at 4 weeks (F1) and at 8 weeks (F2). Patients complete follow-up questionnaires only at their 8 week clinic visit (F2) to reduce burden. In the intervention condition, caregiver questionnaire reminders are paired with a suggestion to review and update their eSNAP visualization, along with a link to do so, prior to questionnaire completion. Every effort is made to obtain questionnaire data within 2 weeks of each scheduled time point, including reminder emails or phone calls or meeting participants in clinic during patient appointments.

At the conclusion of the 8-week study period, participants are debriefed by phone or in person by trained study staff. At this time, waitlisted participants are invited to receive the intervention, completing questionnaires at 4 and 8 weeks to provide some exploratory data on the effect of timing on the intervention. Caregivers who received the intervention will be asked to provide feedback about what they liked and what could be improved.

### Measures

Patient and caregiver participants complete basic demographic and health information questionnaires (including patient function) at baseline. Caregivers complete psychosocial questionnaires at baseline, 4 weeks, and 8 weeks, including measures of burden, distress, and their own and the patients’ health care utilization. Patients complete assessments of distress at baseline and 8 weeks. All data is collected, stored, and managed in secure REDCap databases.

*Caregiver burden* is assessed using the Zarit Burden Interview Short Form [42], a 12-item widely-used measure of burden. Items are summed and higher scores reflect more burden. The scale has been validated in populations of advanced cancer caregivers and has very good internal consistency and discriminative ability [48]. The scale has also been used to identify changes over time [49].

*Distress* is assessed for both patients and caregivers using the Patient Health Questionnaire-8 (PHQ-8) [43] and the General Anxiety Disorder-7 (GAD-7) [44]. The PHQ-8 is an 8-item commonly-used measure of depression based on the Diagnostic and Statistical Manual, 4th edition (DSM-IV) criteria for depressive disorders, and has been shown to have good reliability and validity [43]. The measure has previously been used in studies of cancer patients and caregivers [50]. Each item is rated from 0 to 3, then all 8 items are summed to create a total score ranging from 0 to 24, with higher scores indicating worse depression symptoms; established cutoffs exist [43]. The PHQ-8 is similar to the PHQ-9 (minus a question relevant to suicidality), which has been shown to be sensitive to change [51]. The GAD-7 is a 7 item commonly-used measure of anxiety based on the DSM-IV criteria for Generalized Anxiety Disorder, which has been shown to have good reliability and validity [44]. The GAD-7 has been used extensively in both cancer patients and cancer caregivers (e.g., [50, 52]). Each item is rated from 0 to 3, then all 7 items are summed to create a total score ranging from 0 to 21, with higher scores indicating worse anxiety symptoms; established cutoffs exist [44, 51]. The GAD-7 has been shown to be sensitive to change [53].

*Health Care Utilization* is measured using a self-reported health care utilization questionnaire developed in previous research [54]. Caregivers will be asked to report on their own and the patient’s health care utilization including use of social work or other support services, outpatient appointments, ER visits, and hospitalization, and whether use was related to the caregiver role or stress/ the patient’s cancer.

### Sample size

A power analysis for an independent-groups t-test with a medium effect size (Cohen’s d = 0.5) shows that a final sample of 160 (80 per group) would have Power > .80 with alpha = .05 and a two-tailed test. Based on an estimated 75% recruitment and 15% attrition rate at each follow up, we plan to approach 300 caregivers and enroll 225 to achieve a final sample of 160 dyads completing the 8-week assessment for analyses of hypotheses (80 participants in intervention, and 80 in control conditions).

### Analysis

Our primary end point is 8-weeks; thus our population of interest is caregivers paired with a patient who survives at least 8 weeks. Recruitment will target those early in the care trajectory. Most patients are expected to survive the 8-week study period. The median survival time for patients with glioblastoma, the most common primary malignant brain tumor in adults, is 14.6 months [55]. In the unlikely event patients no longer receive care at Moffitt (transition to palliative/hospice care, change providers, death) participants will not be dropped.

### Missing data

Follow-up data may be missing for a variety of reasons. In general, an intent-to-treat approach has been adopted and study resources will remain available for caregivers and patients whether or not study data is acquired. For example, participants who do not provide data at F1 or who decline navigation will continue to be contacted at F2 to complete questionnaires. Unless the patient and caregiver choose to withdraw from the study, attempts will be made to acquire follow-up data.

### Preliminary analysis

Basic descriptive analyses will be conducted. Chi-square and t-tests will be conducted to determine if the intervention (eSNAP + Caregiver Navigator) or waitlist control groups significantly differ on demographics, patient medical characteristics, or outcome measures at baseline. Any measure with a group differences of *p* < .10 will be controlled for in primary analyses. Group differences and predictors of attrition will be examined using logistic regression. These results will be used to guide interpretation of primary analyses.

### Primary analysis

The primary analysis will evaluate the efficacy of eSNAP + Navigator support intervention on caregiver well-being. This can be performed most simply by using independent-samples t-tests, if randomization is fully successful and missing data are Missing Completely at Random. However, Generalized Estimating Equations (GEE) will be used to test the effects of the intervention at F1 and F2 in models that also include the baseline measure of the outcome variable and, if necessary, potential confounds determined by preliminary analyses. This approach manages missing data under the Missing At Random (MAR) assumption and permits analyses beyond those focusing on the outcome variable at 8 weeks (e.g. determining if intervention has an early impact or if effect matures over time). The primary test will be a planned contrast of the 2 conditions at the 8-week assessment within the entire model. The GEE will also assess (1) intervention differences in the primary outcome averaged across the 4 and 8 week assessments (2), change from 4 to 8 weeks, and (3) group differences in change over time (intervention by time interaction term in the model).

Other analyses will evaluate the interdependent effects of eSNAP + Navigator on patient well-being and health care utilization, hypothesizing that patients with caregivers in the intervention group will report greater well-being and less utilization of health care. The primary outcomes are (1) burden, anxiety, and depression scores as (inverse) measures of well-being, and (2) a set of measures for healthcare utilization: the presence of and number of unplanned outpatient visits, the presence of and number of unplanned hospitalizations, and the number of bed days of care in unplanned hospitalizations. For well-being in patients at 8 weeks, linear regression will be used to assess differences between the intervention arms in the context of any potential confounds. For healthcare utilization, the distributions of the outcome variables are expected to warrant 2-stage and/or Poisson regression analyses to assess intervention differences.

#### Ethical considerations

Ethical approvals have been sought and granted by the Moffitt Scientific Review Committee (MCC 19731) and the Advarra Institutional Review Board (Pro00029204). Informed consent will be obtained from all study participants, in accordance with the Helsinki Declaration. Data will be stored on a secure network, protected by password, and only accessed by researchers involved in this study. This investigation will be carried out in compliance with all human subjects research ethical regulations and guidelines. This study protocol has been registered at clinicaltrials.gov (NCT04268979).

## Discussion

Our protocol will test a novel social support intervention that pairs eSNAP, a web-based social network visualization tool and resource list, and caregiver navigation by telephone to address the support needs of caregivers of patients with primary malignant brain tumor and improve caregiver and patient well-being and health care utilization. This is a high-need population that has not received much attention from research. Our proposed intervention contributes to a family-centered model of care, focusing on patient and caregiver outcomes and facilitating caregiver integration with the health care system. We also reduce the burden of participation in part through the use of technology. We meet all recommendations from a recent joint report from the National Cancer Institute and National Institute of Nursing Research on research and clinical priorities for informal caregiving research [56]. This project is an important step to improving support for caregivers of patients with primary malignant brain tumor, providing appropriate levels of health care utilization, and improving the quality of life for families affected by this disease.

## Data Availability

The datasets generated and/or analysed during the current study will be available from the corresponding author on reasonable request.

## Abbreviations

eSNAP: Electronic Social Network Assessment Program
REDCap: Research electronic data capture
DSM-IV: Diagnostic and Statistical Manual, 4th edition
